# Increased Food Resources Help Eastern Oyster Mitigate the Negative Impacts of Coastal Acidification

**DOI:** 10.3390/ani13071161

**Published:** 2023-03-25

**Authors:** Caroline Schwaner, Michelle Barbosa, Teresa G. Schwemmer, Emmanuelle Pales Espinosa, Bassem Allam

**Affiliations:** School of Marine and Atmospheric Sciences, Stony Brook University, Stony Brook, NY 11790, USA

**Keywords:** oyster, ocean acidification, energy, feeding, metabolism, bioenergetics

## Abstract

**Simple Summary:**

The concentration of CO_2_ in the atmosphere has increased dramatically since the Industrial Revolution because of human activities including burning of fossil fuels. The oceans absorb almost one-third of atmospheric CO_2_, which has led to a decrease in the pH of seawater, a process known as ocean acidification. Animals that build shells from calcium carbonate, such as the eastern oyster, are especially vulnerable to this reduction in pH. Over the last decade, many studies have demonstrated alterations in development and a reduction in shell growth in bivalves exposed to ocean acidification, while less attention has been paid to the mechanisms that enable these animals to survive. This study evaluated the challenges of oysters surviving in low pH conditions and identified potential processes involved in resilience to acidification, such as whether food availability or changes in trophic resources enhance resilience. Findings showed that oysters exposed to low pH had increased energetic demands to cope with acidification. Oysters supplemented with abundant food resources performed much better under acidification (had less mortality and greater growth). Furthermore, oysters demonstrated an ability to alter the food uptake process and optimize energy intake. Results suggest that if oysters have the energetic means to perform adaptive mechanisms, they may be successful under future ocean acidification.

**Abstract:**

Oceanic absorption of atmospheric CO_2_ results in alterations of carbonate chemistry, a process coined ocean acidification (OA). The economically and ecologically important eastern oyster (*Crassostrea virginica*) is vulnerable to these changes because low pH hampers CaCO_3_ precipitation needed for shell formation. Organisms have a range of physiological mechanisms to cope with altered carbonate chemistry; however, these processes can be energetically expensive and necessitate energy reallocation. Here, the hypothesis that resilience to low pH is related to energy resources was tested. In laboratory experiments, oysters were reared or maintained at ambient (400 ppm) and elevated (1300 ppm) *p*CO_2_ levels during larval and adult stages, respectively, before the effect of acidification on metabolism was evaluated. Results showed that oysters exposed to elevated *p*CO_2_ had significantly greater respiration. Subsequent experiments evaluated if food abundance influences oyster response to elevated *p*CO_2_. Under high food and elevated *p*CO_2_ conditions, oysters had less mortality and grew larger, suggesting that food can offset adverse impacts of elevated *p*CO_2_, while low food exacerbates the negative effects. Results also demonstrated that OA induced an increase in oyster ability to select their food particles, likely representing an adaptive strategy to enhance energy gains. While oysters appeared to have mechanisms conferring resilience to elevated *p*CO_2_, these came at the cost of depleting energy stores, which can limit the available energy for other physiological processes. Taken together, these results show that resilience to OA is at least partially dependent on energy availability, and oysters can enhance their tolerance to adverse conditions under optimal feeding regimes.

## 1. Introduction

Coastal estuarine environments, and the marine calcifiers that reside there, are threatened by the absorption of atmospheric CO_2_ that has led to acidification of the seawater (i.e., ocean acidification, OA). This is compounded by nutrient-fueled respiration, freshwater runoffs, and biogeochemical cycling [1,2,3,4]. When CO_2_ dissolves in seawater, it forms carbonic acid, which disrupts the carbonate chemistry equilibrium, resulting in an increase in H^+^, reducing pH. The economically and ecologically important eastern oyster (*Crassostrea virginica*) mineralizes shells from calcium carbonate (CaCO_3_) and is particularly sensitive to changes in carbonate chemistry as biomineralization and dissolution of shell materials are dependent on pH and saturation state (Ω) of CaCO_3_. Consequences of OA can have profound implications for marine ecosystems, with alarming potential socio-economic impacts.

Energy metabolism is critical to an organism’s survival and ability to respond to and tolerate stressful environmental conditions, such as seawater acidification. Adverse conditions can impact the energy balance of an organism by requiring additional energy to maintain homeostasis [5]. During moderate or short-term stress, organisms can compensate for elevated energy by increasing energy intake [5]; however, extreme or long-term exposure could limit energy available for other critical processes, resulting in physiological trade-offs [6,7,8]. An organism’s ability to regulate energy expenditure can determine how it tolerates stressful environmental changes.

Marine organisms show diverse responses to high *p*CO_2_ stress, especially regarding metabolism. Kelley et al. (2017) performed a meta-analysis on the impacts of acidification on metabolic functions and found divergent responses with organisms utilizing varied metabolic strategies to cope with acidification [9]. The meta-analysis revealed trends with respect to life stage, habitat, motility, taxonomy, and differences in acclimation and thus tolerance to OA [9]. Metabolic suppression has been demonstrated in many cases [10,11,12,13,14] and was suggested as an adaptive mechanism for survival under stressful conditions. The other common response of bivalves to OA conditions is metabolic upregulation [15,16,17] to increase physiological processes such as ion transport, acid-base regulation, and calcification. This is not surprising because responses to environmental perturbations are often associated with high metabolic costs of homeostasis. The variability in responses to OA suggests that there are differences in the capacity for different species to acclimate and adapt to OA. As more research emerges, the vast disparities in pH tolerance among and within taxa is further highlighted underlining the need to incorporate more species (and populations) in investigations.

Previous research has shown that the reallocation of the energy budget is a mechanism that could help confer resilience to OA [18,19,20]. For example, the purple sea urchin (*Strongylocentrotus purpuratus*) reallocated energy towards maintenance of ionic homeostasis and protein synthesis to compensate for changes caused by elevated *p*CO_2_ [19,21]; however, this resulted in smaller sea urchin larvae [19,22]. In this case, energy was partitioned toward energetically expensive compensatory processes requiring greater metabolic demand, leaving less energy for growth [19,21,22]. Decreased development and size have also been observed in bivalves under acidification stress [23,24,25,26,27,28,29,30]. Biomineralization in bivalves is energetically expensive, with 31–60% of the total energy budget invested in shell growth [31]. Decreased growth as a response to high *p*CO_2_ might be due to the energetic constraints of organisms under acidification stress. However, elevated energetic demands to sustain calcification can be supported by food availability [32,33,34]. For example, the kinetic constraints on calcification may be offset by adaptive mechanisms such as increasing feeding rates or by eating more nutritious food. Thomsen et al. (2013) demonstrated that the primary factor driving biomass and biogenic CaCO_3_ precipitation of juvenile blue mussels (*Mytilus edulis*) was food availability [34]. High *p*CO_2_ had minimal impact on growth and calcification of the mussels that were exposed to high concentrations of food. Future climatic conditions such as warming, may increase the nutritional quality of algae [35,36] and increase feeding rates of herbivores [37]. The resulting increase in energy transfer from primary producers to secondary consumers could help alleviate detrimental consequences of climate change. However, climate change could also result in a mismatch between larval release and phytoplankton blooms [38], making it more difficult for larvae to meet energetic demands. In addition, studies have demonstrated negative impacts of high *p*CO_2_ on larval feeding activities including delayed initiation of feeding and reduced rates of algae ingestion [39]. Reduced feeding and energy intake in larval bivalves could make them more susceptible to alterations in carbonate chemistry.

Here, we hypothesized that energy is a limiting factor in the success of organisms under OA, and that abundant energy resources might confer resilience. To test this idea, we first established whether exposure to elevated *p*CO_2_ alters energetic demands of the eastern oyster. Increased energy expenditure in larvae and depletion of energy stores (glycogen, lipids) in adults was observed, and it was hypothesized that oysters may implement compensatory mechanisms to offset the changes in the energy budget. Subsequently, investigations focused on whether food availability could help mitigate the impacts of low pH and if oysters can regulate food uptake strategies to maximize energy intake under low pH conditions. Research regarding how the eastern oyster responds to low pH from a metabolic and energetic perspective is rather limited in scope. Our study fills this gap of knowledge by combining multiple physiological assays across different life history stages and different lengths of exposure to provide a comprehensive assessment of the complex interactions between elevated *p*CO_2_ and energy resources in *C. virginica*. In this framework, this is one of the first studies to investigate how food availability and acidification impact eastern oyster D-stage larvae and evaluate oxygen consumption of 2-week-old eastern oyster larvae under low pH. In addition, this is one of the few studies, especially in bivalves, investigating changes to sorting efficiency and food selectivity under low pH. Together, the generated results suggest that if oysters have the energetic resources to enable adaptative mechanisms to respond to high *p*CO_2_, they may be successful under future environments.

## 2. Materials and Methods

### 2.1. Seawater Carbonate Chemistry

#### 2.1.1. Standing System

Eastern oyster (*C. virginica*) larvae cultures were maintained in 43 L tanks submerged in a temperature-controlled water bath (23 °C) to maintain stable temperature between replicates. The target *p*CO_2_ was adjusted by continuously bubbling ambient air for the low *p*CO_2_/high pH or “ambient” treatment (without buffering water to reflect current local environmental conditions) to maintain a target pH of 8.0 (*p*CO_2_ of 400 ppm) into four (*n* = 4) tanks filled with 0.2 µm filtered seawater. For the high *p*CO_2_/low pH or “elevated” treatment, 5% CO_2_ was mixed with air using multi-channel gas proportioners (Cole Parmer; Antalya Scientific; Vernon Hills, IL, USA) and bubbled directly into the seawater of the four replicate tanks to achieve a pH of 7.5 (*p*CO_2_ of 1300 ppm). The low pH level was chosen based on the Intergovernmental Panel on Climate Change (IPCC) predictions in the reduction in pH for the end of the century [40]. Bubbling started 24 h before larvae were added to ensure that target values were reached and pH remained stable.

#### 2.1.2. Flow through System

Adults were held in an open flow through system with water sourced from Old Fort Pond in Southampton, NY (40°53′07.2″ N, 72°26′31.4″ W). For the elevated *p*CO_2_ treatment, water continuously flowed into an acidification chamber where 100% CO_2_ was mixed with air using multi-channel gas proportioners (Cole Parmer) and bubbled to maintain a delta of 0.6 pH units between treatments. This is slightly less than the predicted decrease in pH under the ‘business-as-usual’ scenarios [41]. Water from the chamber then continuously flowed into two replicate s (*n* = 2) corresponding to the elevated treatment using a “downweller” setting that allowed the equilibrated seawater to flow from the top to the bottom compartment of the tanks [42]. For the ambient treatment, water flowed into an aerated head tank where it then continuously flowed into each replicate.

#### 2.1.3. Seawater Parameters

pH was monitored daily using a Durafet III pH probe (Honeywell, Morristown, NJ, USA). Dissolved Inorganic Carbon (DIC) samples were assessed using an EGM-4 Environmental Gas Analyzer ^®^ (PP systems; Amesbury, MA, USA) after acidification and separation of gas phases using a Liquicel ^®^ Membrane (3M; Saint Paul, MN, USA), prior to the introduction of animals and throughout the experiment [42]. Bicarbonate standards were used and certified reference material (Andrew Dickson, Scripps Institution of Oceanography, San Diego, CA, USA) was analyzed for quality assurance with a 99.9% recovery. The following parameters were calculated from pH, temperature, and salinity using the R package *seacarb* [43] and using the first and second dissociation constants of carbonic acid in seawater from Millero, 2010 [44]: *p*CO_2_, Ω _aragonite_, Ω _calcite_, DIC, CO_3_, and total alkalinity (Supplementary Tables S1–S3).

### 2.2. Larval Assays

Brood stock from the Great Atlantic Shellfish Farm, Islip, NY (40°42′19″ N, 73°11′32″ W) was conditioned for spawning [42,45]. After eight weeks of conditioning, a thermal stimulus [45] was used to induce spawning of ripe oysters. Individuals releasing eggs were identified as females (7 females for the April spawn, 6 females for the July spawn) and separated from spawning males (10 males for the April spawn, 6 males for the July spawn) into a separate sea table for holding and egg collection. Sperm was separately collected and mixed (to ensure one male did not fertilize all eggs) and added to eggs. After fertilization (~1 h), embryos were checked for formation of polar bodies and transferred to experimental tanks. Developing larvae were then reared at a stocking density of 10 larvae/mL following methods described in [42,45]. Two separate spawns were performed (different parents but same broodstock or population) and used to evaluate the effect of elevated *p*CO_2_ on respiration (April spawn) and to assess the effect of food availability on larval response to elevated *p*CO_2_ (July spawn).

#### 2.2.1. Micro-Respirometry

After 14 days of exposure to seawater with elevated *p*CO_2_ or ambient (control) conditions, larvae were sampled for respiration assays. Closed respirometry was conducted on larvae randomly sampled from each replicate in each treatment (4 replicates per treatment). Oxygen consumption rates of larvae were measured by two 24-channel SensorDish readers (SDRs; PreSens Precision Sensing, GmbH, Regensburng, Germany) and glass well plates equipped with an optical oxygen sensor spot in each 500 μL well (Loligo Systems, Viborg, Denmark), using protocols outlined in [46]. Each original replicate had three wells represented in the plate: 1 control well to measure microbial respiration (seawater without larvae) and two with larvae (average ~100 larvae/well). The wells were filled completely with seawater, cleared of air bubbles, and sealed with parafilm, silicone, and acrylic sheets. The well plates were submerged in temperature-controlled water baths (23 °C). The system was covered to prevent light interference with the sensors. PreSens SDR software was used to record dissolved oxygen (DO, mg L^−1^) every 15 s. Temperature was measured simultaneously, and data were collected during stable temperature. Data were subset to include a period when temperature was changing by less than 0.1 °C h^−1^ (to account for initial increase in temperature when the sensor dish was turned on). A linear model was fitted to the DO values with respect to time. The slope of each linear model was multiplied by the well volume to obtain oxygen consumption rates, which were converted into units of nmol O_2_ d^−1^. The mean oxygen consumption rates from control wells were subtracted from each larvae-containing well of the same treatment to account for microbial (background) respiration. Respiration was divided by biovolume of live larvae. Larvae were photographed and their surface area was determined using an image analysis software (ImageJ, Version 1.44, NIH, Bethesda, MD, USA) and biovolume was calculated using the equation for a sphere.

#### 2.2.2. Effect of Food Availability on Larvae Resilience to Elevated *p*CO_2_

After 24 h (post fertilization/stocking) in either ambient or elevated conditions (*n* = 4), larvae were further divided into high food and low food treatments. The high food diet represented 100% of the recommended algal quantity per larvae [45,47] or 4000 algal cells/larvae/day (10 larvae/mL; 40,000 cells/mL). The low food condition was 10% or 400 cells/larvae/day (4000 cells/mL). The diet consisted of live *Tisochrysis lutea* cultured in F/2 media. This yielded four different treatments: ambient CO_2_ + high food (AH), elevated CO_2_ + high food (EH), ambient CO_2_ + low food (AL) and elevated CO_2_ + low food (EL). After an additional 24 h (48 h post fertilization) in *p*CO_2_ and food treatment, viability was assessed microscopically. Larvae were sieved (25 µm) and rinsed into a graduated cylinder and a 0.5 mL sample was taken three times and the average number of live and dead larvae was calculated [45]. A sample of larvae was preserved in 1% glutaraldehyde and larval length was determined by measuring the distance between the anterior and the posterior tip of the shell along an axis parallel to the hinge using ImageJ. Length was only measured on larvae viable during the time of preservation, determined by internal complexity and color. A minimum of 100 larvae were measured per replicate.

### 2.3. Adults

Adult oysters (85.55 ± 7.99 mm length; 67.08 ± 10.05 width) were obtained from a commercial source (Frank M. Flower & Sons Inc., Oyster Bay, NY, USA, 40°52′37″ N, 73°31′29″ W). Oysters were scrubbed and cleaned of epibionts and allowed one week of acclimation to laboratory conditions. After acclimation, oysters were moved to the open flow through system described above. Each *p*CO_2_ treatment had two replicates (*n* = 2) with 25 oysters in each replicate. *p*CO_2_ conditions were 7.94 pH and 500 *p*CO_2_ for ambient conditions and 7.3 pH and 3000 *p*CO_2_ for elevated conditions.

#### 2.3.1. Effect of Elevated *p*CO_2_ on Algae Selection

After six months of exposure of adult oysters to high *p*CO_2_, the algae selection experiment was performed to evaluate whether high *p*CO_2_ impacts oyster ability to sort their food particles. Two different species of microalgae were cultured using F/2 media: *Chlamydomonas* sp. (a green algae) and *Rhodomonas lens* (a red algae). These were chosen because of their ability to be clearly distinguished by flow cytometry as well as microscopically on a counting chamber. The algae were grown to the same concentration of algal cells (measured on a hemocytometer ~10^5^/mL). Twenty-four hours before the experiment, oysters were cleaned and held in filtered (0.2 µm) seawater. A 50–50 mixture of the two algae species was made and the ratio was confirmed on the hemocytometer. Fifteen oysters from each replicate in each treatment were individually placed in 3 L tanks and fed a diet of the two algae. The goal was to have at least 10 oysters per treatment actively feeding. Diet samples were collected to assess the proportion of each algae species using a flow cytometer (Becton Dickinson FACSCalibur; BD Biosciences; Franklin Lakes, NJ, USA) as described previously [48]. Pseudofeces produced by oysters during the feeding phase were collected and used to evaluate pre-ingestive selection (i.e., sorting of food particles). Pseudofeces represent material cleared from the water but rejected by oysters before being ingested. To do so, pseudofeces pellets were disrupted using a vortex lab mixer and the proportion of each alga species in the samples was measured using the flow cytometer.

#### 2.3.2. Biochemical Analyses

At the end of the exposure period (10 months), 32 adult oysters were sampled (16 per treatment/8 per replicate). Mantle tissue was collected from each oyster and divided into three pieces for downstream analyses (~50 mg per piece). Biochemicals measured included glycogen, lipids, and proteins [8]. Mantle tissue was selected as this tissue is an important area for bivalves to store energy for physiological processes [49,50]. Glycogen was measured using Sigma-Aldrich Glycogen colorimetric assay kit (Sigma-Aldrich, St. Louis, MO, USA) according to the manufacturer’s protocol. Lipids were measured gravimetrically after solvent evaporation following [51]. Lastly, proteins were measured using the Pierce BCA protein assay reagent kit (Pierce, Rockford, IL, USA) following manufacturer’s protocol.

### 2.4. Statistical Analyses

Statistical analyses were conducted using R version 4.2.0. Assumptions of a normal distribution and homoscedasticity were confirmed using the Shapiro–Wilk and Bartlett’s tests, respectively. All results were deemed significant at ɑ ≤ 0.05.

The tank replicates per *p*CO_2_ treatment were designated as the experimental units (*n* = 4 for larvae or *n* = 2 for adults). Based on recommendations by Cornwall and Hurd (2016) [52] we set *p*CO_2_ treatment as a fixed effect and replicate (tank) as a random effect nested within *p*CO_2_ treatment. For the respiration data and the concentration of glycogen, lipid, and protein, we performed a nested *t*-test to detect significant differences. Respiration data were transformed using exponential transformation to meet the assumptions of the test. For larval lengths a nested ANOVA was performed. Post hoc Tukey tests were performed for pairwise comparisons as needed (or when significant main effects were detected). Viability (% mortality) was compared using G-test of independence and pairwise post hoc G-tests. For the algae selectivity data, a series of goodness-of-fit tests were performed on raw counts to determine that replicates within each treatment were homogenous and to test the null hypothesis that within each treatment the proportion of each alga was the same in diets and pseudofeces [48]. The sorting efficiency (SE) index was calculated to determine particle selection [48]. The equation for sorting efficiency is SE = 1 − (P/D), where P and D represent the proportion of the algae of interest in pseudofeces (P) and diet (D). If SE is positive, this indicates algae was preferentially ingested, while a negative value represents rejection. After calculating sorting efficiency, the values were compared to zero using a *t*-test. The null hypothesis was that the sorting efficiencies are equal to zero (no selection). A second *t*-test was performed to compare SE of alga between different *p*CO_2_ treatments.

## 3. Results

### 3.1. Effect of Elevated pCO_2_ on Larvae Respiration

**Figure 1:**
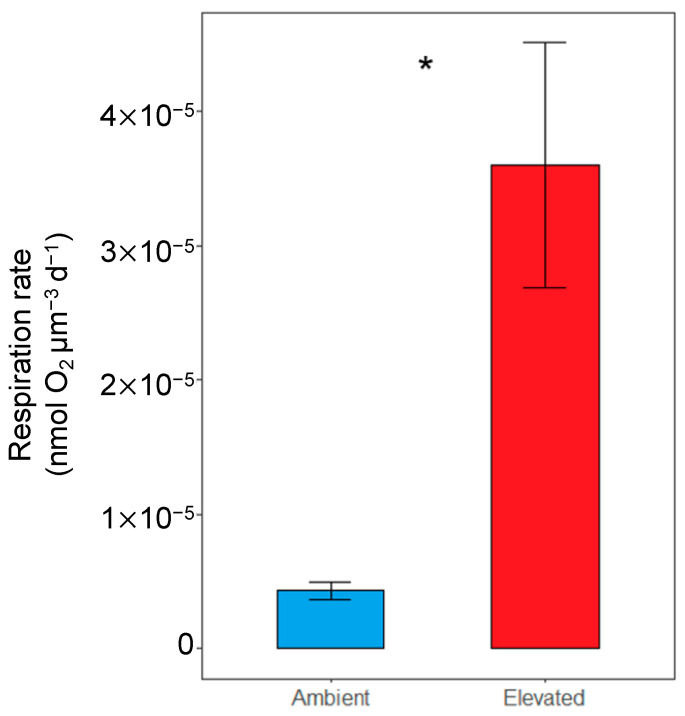
Oxygen consumption (mean ± standard error) normalized to biovolume (µm^3^) of oyster larvae reared under ambient (control) and elevated (acidified) conditions. The asterisk denotes significant difference (nested *t*-test *n* = 4, *p* < 0.01).

Oxygen consumption of larvae from ambient *p*CO_2_ and elevated *p*CO_2_ was compared to determine whether larvae in elevated *p*CO_2_ conditions had increased metabolic demand. Larvae from seawater with elevated *p*CO_2_ had significantly greater respiration (3.6 × 10^−5^ nmol O_2_ µm^−3^ d^−1^; *n* = 4, *p* < 0.01; nested *t*-test; Figure 1) than larvae from seawater with ambient *p*CO_2_ (4.32 × 10^−6^ nmol O_2_ µm^−3^ d^−1^), supporting that the metabolic demand is higher for larval oysters cultured under elevated *p*CO_2_ conditions.

### 3.2. Effect of Food Availability on Larvae Resilience to Elevated pCO_2_

**Figure 2:**
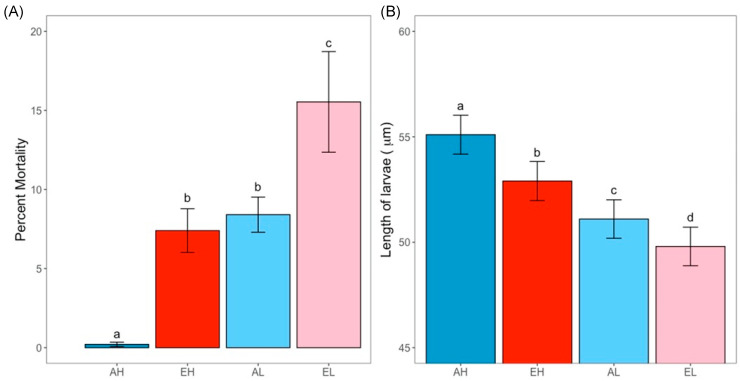
Percent mortality (**A**) and length (**B**) of oyster larvae held at different *p*CO_2_ and food conditions (mean ± standard error). The different letters (a through d) denote significant differences between treatments (G-tests with pairwise post hoc tests for A, and nested ANOVA and Tukey post hoc tests for B). Labels along the *x*-axis denote treatment codes with the first letter representing *p*CO_2_ condition (A = ambient, E = elevated) and the second letter designating food level (H = high, L = low).

Both *p*CO_2_ and food level had an impact on oyster survivorship (G-test; post hoc pairwise G-tests; *p* < 0.05; Figure 2A). Larval oysters in ambient conditions with high food had negligible mortality (0.2%) which was significantly less than any other group. Oysters in elevated *p*CO_2_ with high food had significantly greater mortality (7.4%) than their ambient counterparts. Within elevated *p*CO_2_, oysters with the low feeding regime had the highest mortality (15.5%) which was significantly greater than any other group. Interestingly, there was no difference in survivorship between oysters grown under elevated *p*CO_2_ and high food and those grown under ambient *p*CO_2_ and low food conditions. Growth results (using length as a proxy for growth) had similar overall trends as mortality although all treatments were significantly different from each other. Both high food conditions (ambient and elevated *p*CO_2_) were significantly larger than their low food counterparts (nested ANOVA; Tukey post hoc tests; Figure 2B). Oysters from the ambient *p*CO_2_ and high food were significantly larger (55.1 ± 0.9 µm) than those from all other treatments, followed by those grown under elevated *p*CO_2_ and high food conditions (52.9 ± 0.9 µm), ambient *p*CO_2_ and low food conditions (51.1 ± 0.9 µm), and finally by oysters raised under elevated *p*CO_2_ and low food conditions (49.8 ± 0.9 µm). Overall, findings show that oysters had less mortality and were larger in size when food was abundant in high *p*CO_2_ conditions compared to when food was scarce.

### 3.3. Effect of Elevated pCO_2_ on Algae Selection

**Figure 3:**
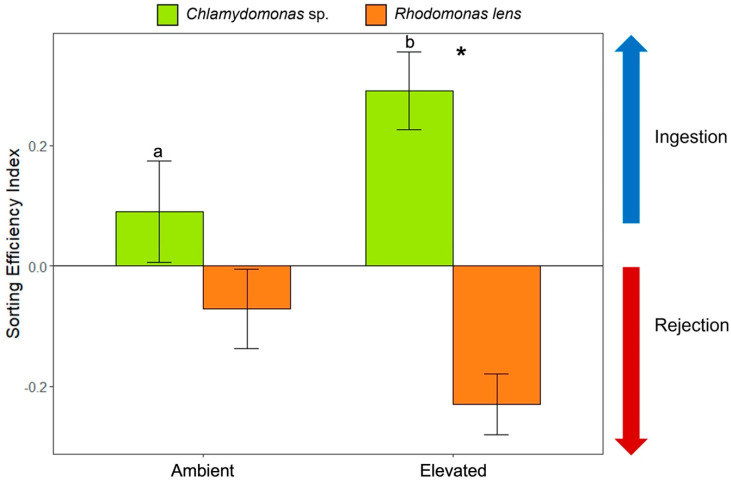
Sorting efficiency (mean ± standard error) in oysters maintained under ambient or elevated *p*CO_2_. Asterisk denotes significant sorting (elevated *p* = 0.0007 and ambient *p* = 0.15). Different letters (a and b) denote significant differences between treatments (two sample *t*-test, *p* = 0.001).

Preference between the two algae *Chlamydomonas* sp. and *Rhodomonas lens* was tested to determine if oysters become more selective under elevated *p*CO_2_ conditions as a strategy to optimize energy gains. Algae selectivity of adult oysters maintained in ambient *p*CO_2_ was not significantly different than 0 (*p* = 0.15; Figure 3). In other words, oysters did not show a preference for one alga over the other. In contrast, oysters became significantly more selective under elevated *p*CO_2_ conditions (sorting efficiency index significantly different than 0, *p* = 0.0007) by preferentially ingesting *Chlamydomonas* sp. and rejecting *R. lens* under acidified conditions. The sorting efficiencies were also compared between treatments and results showed significantly higher sorting efficiencies in oysters grown under acidified conditions as compared to ambient conditions (two-sample *t*-test, *p* = 0.001; Figure 3).

### 3.4. Biochemical Analyses

**Figure 4:**
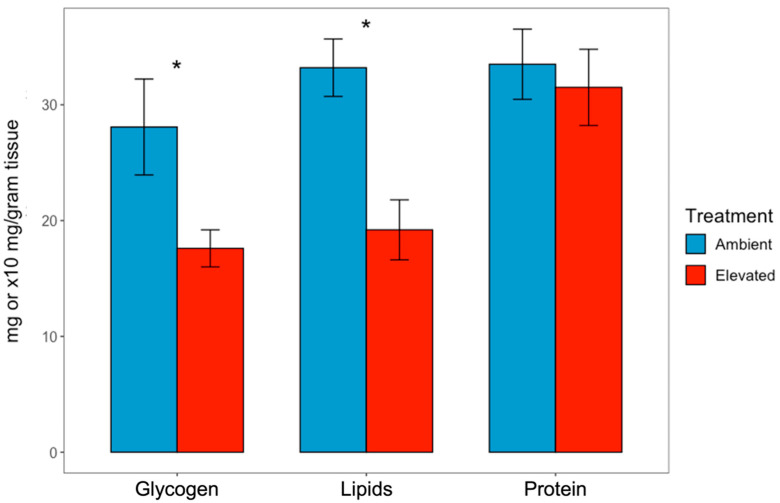
Concentrations of glycogen (mg/g tissue), lipid (mg/g tissue) and protein (×10 mg/gram tissue) in mantle tissue from oysters held in ambient or elevated *p*CO_2_ (mean ± standard error, asterisk denotes significant differences between treatments, nested *t*-test, *p* < 0.05, *n* = 2).

The concentration of glycogen, lipid, and protein in mantle tissues of oysters cultured under ambient and elevated *p*CO_2_ conditions was compared to evaluate how acidification affects energy reserves. Glycogen concentration of mantle tissue from oysters in the elevated *p*CO_2_ condition (17.6 ± 1.6 mg/g tissue) was significantly lower than that derived from oysters in the ambient condition (28.08 ± 4.1 mg/g tissue; nested *t*-test, *n* = 2; *p* <0.05; Figure 4). Lipid concentration from oysters in the elevated *p*CO_2_ condition (19.2 ± 2.6 mg/g tissue) was also significantly less than that of oysters in the ambient condition (33.2 ± 2.5 mg/g tissue). There were no differences in protein concentration from oysters in the elevated *p*CO_2_ as compared to the ambient condition (314.5 ± 33 and 335.3 ± 30 mg/g tissue, respectively; *p* > 0.05).

## 4. Discussion

This investigation was designed to probe energetic processes associated with resilience to high *p*CO_2_ environments in *C. virginica*. Metabolic responses to elevated *p*CO_2_ conditions is a polarizing area of research, with variability between taxa, species, life stages, and populations from different geographic locations. The work presented here investigates various energetic responses to high *p*CO_2_ across a range of life history stages of the eastern oyster. Increased energy expenditure was observed in larval oysters in response to elevated *p*CO_2_ which inspired further investigation of compensatory mechanisms to enhance energy intake. Increasing larval feeding in aquaculture settings is a possible strategy that hatchery managers could adopt to mitigate impacts of high *p*CO_2_ in their source water. Thus, a food limitation experiment was conducted and showed that increased food resources mitigate the negative impacts of high *p*CO_2_ during initial development stages of larvae. Adult oysters are known to preferentially ingest more nutritious algae over non-nutritive algae and debris present in seawater via an elaborate process called particle sorting. Results presented here showed that adult oysters increased food selectivity under elevated *p*CO_2_, likely to optimize energy gain and fight acidification stress. The negative impacts of elevated *p*CO_2_ do not always manifest in observable phenotypic changes or death, but if stressed animals are depleting energy reserves during chronic exposure to elevated *p*CO_2_, there could be physiological trade-offs including costs to other important physiological processes such as gametogenesis or immune responses. In this framework, results also showed a significant decrease in energy storage among adult oysters exposed to chronic acidification stress, highlighting the energetically costly nature of resilience mechanisms.

### 4.1. Enhanced Metabolic Demand of Oyster Larvae under Elevated pCO_2_

The 14-day-old larvae maintained in elevated *p*CO_2_ conditions consumed a significantly greater amount of oxygen than those cultured under ambient conditions (over 8 times more). In theory, oxygen consumption should increase with oyster size [53]. However, we saw that the larvae exposed to elevated *p*CO_2_ that consumed more oxygen were significantly smaller, contradicting this. Stumpp et al. (2011) reported an increased metabolic rate and smaller size of sea urchins (*Strongylocentrotus purpuratus*) under elevated *p*CO_2_ compared to controls [22]. The increased respiration affected the scope for growth and was correlated to the observed urchin growth. The decrease in scope for growth during early development suggests that maintenance needs become increasingly energy-consuming with elevated *p*CO_2_ thus larvae in elevated *p*CO_2_ face greater energetic demands to supply vital processes [22]. This could have impacts on long-term ability to upregulate other physiological processes (such as ion regulation and biomineralization) in response to low pH. The increased respiration observed in the oyster larvae is indicative of a similar stress response as the sea urchins under elevated *p*CO_2_ conditions, i.e., they are expending more energy to survive the adverse environment. A metabolic increase under elevated *p*CO_2_ is indicative of upregulating processes that are energetically costly such as acid-base balance, calcification, or apoptosis (reviewed in [9]). Lower metabolic rates are usually associated with lower concentrations of ion transport proteins [54], reducing the ability to regulate acid-base balance. Increasing metabolism is associated with enhanced tolerance to high *p*CO_2_ because organisms can have higher ion and acid base regulation, protein synthesis, and growth [17,20,21]. For example, Pansch et al. (2014) reported that energy availability can mediate the ability of barnacles to withstand moderate CO_2_ stress [20]. Pan et al. (2015) also found that sea urchin larvae reallocate energy to support increased protein synthesis and ion transport under low pH conditions [21]. Using proteomics, Dineshram et al. (2015) demonstrated an upregulation of proteins involved in energy production and metabolism in response to low pH in larval *Crassostrea hongkongensis* [55]. The authors reported that this supports their hypothesis that increased metabolic processes contribute to normal larval growth and metamorphosis under low pH conditions [55]. In addition, increased metabolism was found in the mussel *Mytilus edulis* [16], the gastropod *Concholepas concholepa* [15], and the surf clam *Spisula solidissima* [17] in response to high *p*CO_2_. Metabolism was also previously shown to be upregulated in juvenile eastern oysters under high *p*CO_2_ conditions [56,57]. While our findings suggest upregulation of metabolically costly processes to compensate for elevated *p*CO_2_, one needs to consider that this response might be due to an initial acclimatization and might differ with length of exposure and life stage. Opposite trends of reduced respiration were found in bivalve species as a response to CO_2_ stress such as the hard shell mussel *Mytilus coruscus* [13], blood clam *Tegillarca granosa* [14], and the noble scallop *Chlamys nobilis* [11]. However, we should consider that these studies were conducted on animals at different life stages and geographic locations. Metabolic suppression is achieved by stopping energetically expensive processes, and as such, can come with associated costs. For example, Zhao et al. (2017) found reduced calcification and shell corrosion from insufficient energy supply, from metabolic suppression, in the blood clam exposed to low pH conditions [14]. Under chronic exposure to elevated CO_2_, metabolic suppression may have associated costs.

### 4.2. Abundant Food Helps to Mitigate the Impacts of Elevated pCO_2_ on D-Stage Larvae

Because larvae require more energy under elevated *p*CO_2_, they need to compensate for this increased demand. Based on the results of the respiration assays, we tested the hypothesis that resilience to high *p*CO_2_ is related to the availability of food resources. Although we were unable to perform the food assay in parallel to the respiration assay, larvae used derived from the same broodstock and had similar environmental histories. Larvae performed better (increased viability, greater growth) under elevated *p*CO_2_ conditions with abundant food, supporting the hypothesis that resilience to elevated *p*CO_2_ is related to energy. A food limitation assay was performed, in which D-stage oysters were exposed to different *p*CO_2_ conditions as well as different concentrations of food. Findings showed that both *p*CO_2_ and lack of food are stressors, and the stress of food limitation and low pH are additive. Under poor food conditions and high *p*CO_2_, the larvae had the greatest apparent mortality and were the smallest. While under high *p*CO_2_ conditions with abundant food, they had less apparent mortality and were significantly larger. These results may suggest that food limitation during early development stages of oysters exacerbates underlying stresses that may arise from a high *p*CO_2_ seawater environment. Given that lipid reserves from the egg likely provide energy to oyster larvae during initial development [58], the significant impact of exogenous feed availability on growth and survival at this stage is striking.

Other studies have shown similar results, supporting our findings that food availability might confer resistance to low pH. For example, Sanders et al. (2013) suggested that when food is abundant, bivalves are more tolerant to changes in pH [59]. Oyster larvae have been shown to be less tolerant to starvation under elevated *p*CO_2_ conditions [60,61]. A meta-analysis by Ramajo et al. (2016) showed that food supply confers resistance to OA [62]. The authors concluded that when the energy supply is high enough organisms can upregulate processes such as ion transport and calcification or increase the pH in hemolymph or the extrapallial fluid (calcification fluid located between the mantle and the shell), which are metabolically demanding processes. Food supply can modulate responses by providing the energetic means to perform adaptive mechanisms to mitigate the impacts of low pH. Enhanced food availability increased the resilience of fish [63], barnacles [20], mussels [34], juvenile scallops [64], and larval Olympia oysters [65] to low pH. In addition, some studies have reported an increase in clearance rate or feeding activity under low pH. For instance, the Chilean scallop *Argopecten purpuratus* [64] and mussel *Perumytilus purpuratus* [66] both increased feeding to optimize energy intake under low pH. However, other studies showed opposite trends, with decreased clearance, meaning high *p*CO_2_ elicited suppression of feeding. For example, clearance was decreased in the blood clam *Tegillarca granosa* [14], Manila clam *Ruditapes philippinarum* [67], surf clam *Spisula solidissima* [17], European clam *Ruditapes decussatus* [68], mussels *M. coruscus* [13], *Perna viridis* [11], *Mytilus chilensis* [69], scallop *C. nobilis* [11], brooding flat oyster larvae [60], and a snail *C. concholepas* larvae [70]. A deficient energy supply limits the ability of an organism to respond to acidification stress and can make it more vulnerable to elevated *p*CO_2_.

Lutier et al. (2022) found a relationship between pH level and feeding in the Pacific oyster and identified a tipping point of a pH of 6.9 [71]. It is important to consider a more extreme pH level may lessen the impact of increased food on resilience. Differences in feeding might be associated with several factors such as life history stage, impairment of feeding organs, spawning vs. brooding, food concentrations in natural environments, size, and co-occurring stressors such as temperature. Calculation of feeding rates in larvae was outside the scope of this study, but future investigations could shed light on how this related to energy intake.

### 4.3. Adult Oysters in the Elevated pCO_2_ Condition Had Greater Selectivity and Sorting Efficiency

Results demonstrated the importance of abundant food, and to further investigate the idea that food uptake is related to tolerance to high *p*CO_2_, selectivity of food was investigated. The hypothesis that oysters become more selective of algae under high *p*CO_2_ conditions to optimize energy intake was tested. At the adult stage, farmed oysters would not receive supplemental feeding from laboratory cultures, so another strategy of increasing energy was investigated for adults. Increased food selectivity has been demonstrated in other organisms as a response to high *p*CO_2_ [72,73]. As suspension-feeders, oysters are exposed to a myriad of particles including non-nutritive material and debris [74]. They have a mechanism to allow them to enhance nutritive value and optimize energy gain instead of blindly ingesting all suspended materials present in seawater. This discrimination among particles, specifically out of a mix of microalgae of similar sizes and shapes, has been previously demonstrated [75,76,77]. It is known that they can ingest the most nutritious food, but this study investigates if this selection process was enhanced under elevated *p*CO_2_ conditions. Results showed that, while *Chlamydomonas* sp. was always preferentially ingested over *R. lens*, this selection was very weak (not significant) under ambient *p*CO_2_, and only became significant under the elevated *p*CO_2_ treatment, underlining oyster’s ability to regulate the sorting efficiency and enhance energy gains under elevated *p*CO_2_ conditions. Previous studies reported that *Chlamydomonas* sp. represent a good nutritional source for multiple bivalve species. For instance, Foe and Knight (1986) showed strong tissue growth in the clam *Corbicula fluminea* fed with diets containing *Chlamydomonas* sp. [78]. Another study showed that *Chlamydomonas* sp. was preferentially ingested and was better digested by larval conchs (*Strombus gigas*) as compared to *Thalassiosira fluviatilus* [79]. On the other hand, *R. lens* has been shown to be poorly captured by larval mussel *Mytilus trossulus* [80]. Future studies exploring why *Chlamydomonas* sp. was selected over *R. lens* might further elucidate mechanisms of selection, especially selectivity under environmental stress, and how this selection specifically benefits oysters under elevated *p*CO_2_. Despite these limitations, it is clear that the preferential ingestion of *Chlamydomonas* sp. was significantly greater under elevated *p*CO_2_ conditions. Vargas et al. (2013) reported differences in selectivity of algae in larval gastropods which they suggested might be due to negative impacts on larval growth and development that led them to preferentially ingest smaller particles [64]. Adult oysters feeding organ sizes were not compared, but there were no differences in size of adults between treatments. While CO_2_ can have direct impacts on bivalve physiology it can also have indirect impacts by decreasing nutritional quality of algae species, including *Isochrysis galabana* (commonly used in hatcheries) [81]. Duarte et al. 2015 also demonstrated a reduction in protein and organic content of algae species and changes in algal palatability to amphipods under elevated *p*CO_2_ [73].

The increased selectivity seen here, likely represents a strategy to maximize energy and nutrient uptake and thus offset energy deficits that might be created by upregulating energetically expensive processes needed to tolerate CO_2_ stress. It appears that adult oysters can compensate for enhanced metabolism under elevated *p*CO_2_ conditions; however, if food is limited or the quality of food particles is poor, they might not be able to meet the energetic demands of surviving under acidification stress.

### 4.4. Depletion of Energy Reserves after 10 Months of Adult Oyster Exposure to Elevated pCO_2_

Glycogen and lipids represent the main energy stores available for metabolic and fitness related activities. The ability to store energetic substrates is a trait that can help confer resistance to environmental stressors. Prolonged exposure to stressful environmental conditions could result in breaking down of energy reserves required for reproductive success such as glycogen, lipids, and proteins, which can be used for maintenance needs instead of being allocated for reproduction [8]. Under periods of adverse environmental conditions, there can be a mismatch between energy demand and supply from the environment which leads to depletion of energy storage molecules. This depletion could have direct effect such as reduced gametogenesis, lower recruitment potential, and could negatively affect maternal provisioning. Sustained depletion of reserves can lead to mortality. Here, there was significant reduction in lipid and glycogen concentrations, but not protein. Glycogen is the primary energy reserve in bivalves [82] and is the most dynamic, so it is not surprising that there were significant differences between control and acidification stressed individuals. The lack of differences between protein concentration reported here in oysters is not surprising since protein is more stable than other reserve molecules [83].

The reduction in energy reserves can also have indirect effects such as a decrease in organism’s ability to tolerate prolonged exposure to adverse environmental conditions, making them more susceptible to other stressors. For example, reduced energy reserves mean less resources are available for other physiological functions such as immune responses to pathogens. While compromised immunity was not investigated in this study, it has been demonstrated as a potential cost of tolerating high *p*CO_2_ in bivalves [42,84,85]. Depletion of energy reserves because of exposure to high *p*CO_2_ environments has been demonstrated in other species. For example, exposure of the polychaete *Diopatra neapolitana* to elevated *p*CO_2_ resulted in a significant decrease in glycogen and protein as compared to worms held under control conditions [86]. Similarly, glycogen was significantly reduced under elevated *p*CO_2_ in the Yesso scallop *Patinopecten yessoensis* [87] and *Crassostrea gigas* [88]. Finally, lipids were depleted in *Saccostrea glomerata*, *C. gigas* [89] *Mercenaria mercenaria* [90], and *C. virginica* larvae [91] maintained at elevated *p*CO_2_ conditions. The adult oysters used in our study were exposed to chronic acidification stress for 10 months, without supplemental feeding as they only received algae in the raw seawater, which changes seasonally. It remains unclear how these oysters would have responded to acidification stress if the experiment was extended further (e.g., through a reproductive cycle for example), or if they were actively fed using cultured algae to evaluate whether supplemental feeding would have prevented depletion of energy reserves, lessening the impacts of elevated *p*CO_2_.

## 5. Conclusions

This study provides evidence to support the hypothesis that energy resources derived from food represent a limiting factor in the success of oyster response to elevated *p*CO_2_. Oysters exposed to elevated *p*CO_2_ displayed increased respiration at the larval stage and mobilization of energy reserves to cope with acidification stress after chronic exposure. This reallocation of energy towards processes vital for resilience appears to represent an adaptive strategy to tolerate alterations in the carbonate chemistry of seawater. Larval oysters supplemented with abundant food resources performed much better under acidification stress. Further, results demonstrated that adult oysters can adjust their food uptake strategies under elevated *p*CO_2_ to increase particle sorting and optimize energy gains. These results underline the complexity of predicting how marine calcifiers would respond to high *p*CO_2_ under future climate conditions, especially when food availability (quantity and quality) is predicted to dramatically change under projected temperature and *p*CO_2_ conditions. Overall, if oysters have the energetic means to perform adaptive mechanisms to mitigate the impacts of low pH, they might be successful under future climate regimes. Further potential for adaptation to OA in the eastern oyster comes from the extraordinary genetic diversity of the species that may allow the selection of resilient genotypes capable of sustaining robust populations under future climate conditions. Future research can and should integrate these genetic considerations in ecophysiological investigations to provide a complete picture of the potential mechanisms associated with resilience to acidification in the eastern oyster.

## Data Availability

Data is available upon request.

## Supplementary Materials

The following are available online at https://www.mdpi.com/article/10.3390/ani13071161/s1, Table S1: Seawater carbonate chemistry from respiration assay; Table S2: Seawater carbonate chemistry from food limitation assay; Table S3: Seawater carbonate chemistry of flow through system housing adults used in biochemical analyses and sorting.
