# Supplementary material for: Increased Food Resources Help Eastern Oyster Mitigate the Negative Impacts of Coastal Acidification
Source: Animals (Basel). 2023 Mar 25;13(7):1161. doi: 10.3390/ani13071161 (PMC10093323; doi:10.3390/ani13071161)
Supplement: Supplementary file 1 [file animals-13-01161-s001.zip › animals-2230561-supplementary.pdf]

Table S1. Seawater carbonate chemistry values (±) SD for respiration assay

	**Ambient *p*CO_2**	**Elevated *p*CO_2**
**Temperature °C**	23.49±2.37	23.49±2.42
**pH_T**	7.92±0.06	7.57±0.02
***p*CO_2 (μatm)**	458.44±26.57	1138.92±269.75
**Ω Ca**	2.76±0.74	1.30±0.26
**Ω Ar**	1.77±0.47	0.83±0.16
**TCO_2 (μmol L^{-1})**	1641.81±349.39	1741.85±449.15
**CO_3^{2-} (μmol L^{-1})**	108.47±30.34	51.12±10.66
**Total Alkalinity (μmol L^{-1})**	1789.68±383.12	1783.31±448.90
**Salinity**	27.33±0.72	27.33±0.72

Table S2. Seawater carbonate chemistry values (±) SD for food limitation assay

	**Ambient *p*CO_2**	**Elevated *p*CO_2**
**Temperature °C**	21.84±0.06	21.63±0.11
**pH_T**	8.14±0.08	7.49±0.09
***p*CO_2 (μatm)**	322.57±78.74	1600.23±346.23
**Ω Ca**	5.20±0.83	1.28±0.24
**Ω Ar**	3.36±0.54	0.83±0.15
**TCO_2 (μmol L^{-1})**	1981.30±35.41	2060.09±44.85
**CO_3^{2-} (μmol L^{-1})**	209.07±33.58	51.61±9.49

**Total Alkalinity (μmol L^{-1})**	2268.11±67.95	1783.31±448.90
**Salinity**	30±0	30±0

Table S3. Seawater carbonate chemistry values (±) SD for flow through system housing adults used in biochemical analyses and sorting assay

	**Ambient pCO_2**	**Elevated pCO_2**
**Temperature °C**	23.44±3.34	23.43±3.21
**pH_T**	7.94±0.02	7.30±0.43
**pCO_2 (μatm)**	521.53±48.68	3061.44±0.66
**Ω Ca**	3.45±0.30	1.03± 0.76
**Ω Ar**	2.23±0.22	0.66 ±0.48
**TCO_2 (μmol L^{-1})**	2036.10±161.36	2225.38±374.77
**CO_3^{2-} (μmol L^{-1})**	142.36±18.12	41.63±29.87
**Total Alkalinity (μmol L^{-1})**	2227.00±188.47	2179.65±231.50
**Salinity**	27.03±2.86	27.40±3.39
